# Efficacy of a single injection compared with triple injections using a costoclavicular approach for infraclavicular brachial plexus block during forearm and hand surgery: A randomized controlled trial

**DOI:** 10.1097/MD.0000000000022739

**Published:** 2020-10-23

**Authors:** Mi Geum Lee, Wol Seon Jung, Doo Yeon Go, Sung Uk Choi, Hye Won Shin, Yun Suk Choi, Hyeon Ju Shin

**Affiliations:** a Department of Anesthesiology and Pain Medicine, Gachon University College of Medicine, Gil Medical Center, Incheon; b Department of Anesthesiology and Pain Medicine, Korea University College of Medicine, Anam Hospital, Seoul; c Department of Anesthesiology and Pain Medicine, JeJu National University College of Medicine, Jeju Hospital, Jeju, Republic of Korea.

**Keywords:** brachial plexus block, costoclavicular approach, infraclavicular block, triple injection, ultrasound

## Abstract

**Objectives:**

It was recently proposed that a costoclavicular (CC) approach can be used in ultrasound (US)-guided infraclavicular brachial plexus block (BPB). In this study, we hypothesized that triple injections in each of the 3 cords in the CC space would result in a greater spread in the 4 major terminal nerves of the brachial plexus than a single injection in the CC space without increasing the local anesthetic (LA) volume.

**Methods:**

Sixty-eight patients who underwent upper extremity surgery randomly received either a single injection (SI group, n = 34) or a triple injection (TI group, n = 34) using the CC approach. Ten milliliters of 2% lidocaine, 10 mL of 0.75% ropivacaine, and 5 mL of normal saline were used for BPB in each group (total 25 mL). Sensory-motor blockade of the ipsilateral median, radial, ulnar, and musculocutaneous nerves was assessed by a blinded observer at 5 minutes intervals for 30 minutes immediately after LA administration.

**Results:**

Thirty minutes after the block, the blockage rate of all 4 nerves was significantly higher in the TI group than in the SI group (52.9% in the SI group vs 85.3% in the TI group, *P* = .004). But there was no significant difference in the anesthesia grade between the 2 groups (*P* = .262). The performance time was similar in the 2 groups (3.0 ± 0.9 minutes in the SI group vs 3.2 ± 1.2 minutes in the TI group, respectively; *P* = .54).

**Discussion:**

The TI of CC approach increased the consistency of US-guided infraclavicular BPB in terms of the rate of blocking all 4 nerves without increasing the procedure time despite administering the same volume of the LA.

## 1 Introduction

Currently, ultrasound (US)-guidance are being used to assess anatomical position rapidly, to guide the block needle safely, and to confirm the correct distribution of local anesthetic (LA) accurately.^[1,2]^

A conventional (paracoracoid) approach has been used for US-guided infraclavicular brachial plexus blocks (BPBs), in which the LA is deposited dorsally to the axillary artery in the lateral infraclavicular fossa, and the BPB is deep (4–6 cm), perivascular (target point is dorsal to the axillary artery), and requires a large volume of LA (up to 35–40 mL).^[3–5]^ Therefore, it can be technically challenging, despite its efficacy and safety, compared with the supraclavicular or axillary block, which appear to be preferable.

Li et al^[6]^ recently described a new costoclavicular (CC) approach, in which the brachial plexus is targeted immediately caudal to the midpoint of the clavicle in the CC space. In this space, the 3 cords of the brachial plexus are tightly clustered together laterally to the axillary artery. Thus, the approach can be superficial, away from the axillary artery, and requires a relatively small volume of LA (20–25 mL).^[3,6,7]^

Songthamwat et al^[7]^ reported that the CC approach induces a faster onset of a sensory blockade than the conventional approach, even with 25 mL of the LA. They performed the CC approach with a single injection, which was effective for induction of surgical anesthesia for all patients. However, the rate of blockage of all 4 nerves was not significantly high, with a complete sensory blockade rate of 50% 30 minutes after the block. The 3 cords of the brachial plexus are widely distributed laterally to the axillary artery, even though they are tightly clustered together.^[7]^ Therefore, we considered that performing a single injection targeted at the center of the 3 cords could increase the chance of uneven spreading of LA.

Considering this CC topography, we hypothesized that injections in each of the 3 cords, using one-third of the injection volume for each cord, would result in an increased rate of blockage of all 4 nerves compared with a single injection, without an increase in the LA volume.

## 2 Materials and methods

### 2.1 Study population

Written informed consent was obtained from all patients after the study protocols were approved by the Korea University's institutional ethics committee (2019AN0532) and the trial was registered in the University Hospital Medical Information Network (UMIN) Clinical Trials Registry (UMIN000038958). This study was performed in accordance with the Consolidated Standards of Reporting Trials (CONSORT) 2010 checklist.

**Figure 1:**
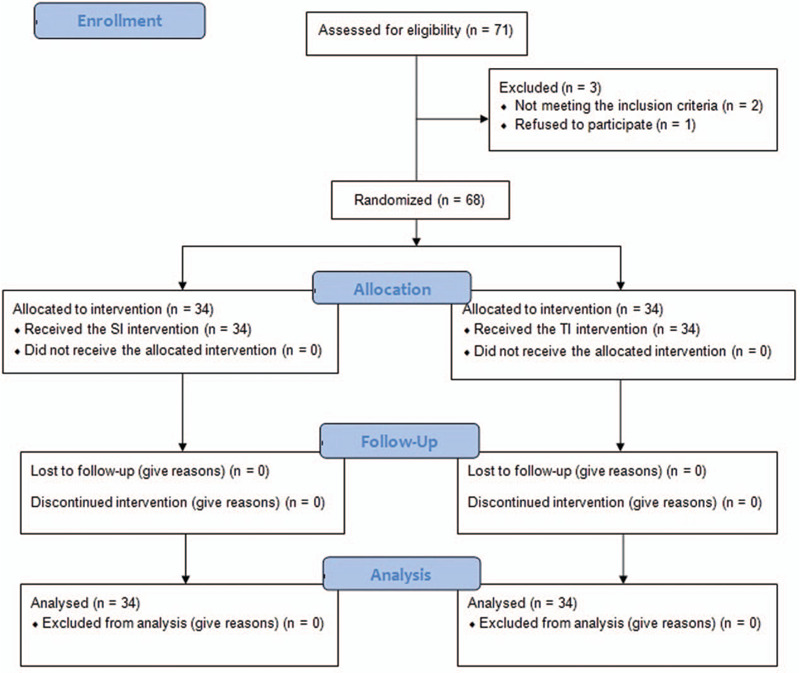
Patients’ enrollment algorithm. SI = single injection, TI = triple injection.

Sixty-eight patients scheduled for surgery of the forearm and hand, were enrolled in the study. The patients were aged 18 to 80 years and had an American Society of Anesthesiologists (ASA) physical status of I–III. The exclusion criteria included preexisting neuropathy in the operated limb, coagulation disorders, known allergy to local anesthetic, local infection at the puncture site, chronic obstructive pulmonary disease or respiratory failure, pregnancy, breast-feeding, body mass index ≥35 kg/m^2^, failure to cooperate, and refusal to participate. We conducted a randomized controlled parallel-group study (Fig. 1).

The patients were randomly assigned to either the single injection group (SI group, n = 34) or the triple injection group (TI group, n = 34) using a random integer set generator (http://www.random.org/). The ratio of allocation was 1:1. A researcher who was not involved in performing the block generated the randomization set and enrolled the participants. All the procedures were conducted at Anam Hospital, Korea University College of Medicine, Seoul, Korea, from December 2019 to March 2020.

### 2.2 Procedures

All infraclavicular BPBs were performed in the anesthesia procedure room, approximately 1 hour before the scheduled surgery. On arrival, supplemental oxygen and standard monitoring (noninvasive blood pressure, electrocardiogram, and pulse-oximetry) were applied, and a time-out procedure was performed. Intravenous premedication (50 μg fentanyl and 1 mg midazolam) was administered to all patients. All blocks were performed by an experienced anesthesiologist (SHJ).

The patients were placed in the supine position, with their ipsilateral arm abducted to 90° and palms facing the ceiling. The patient's head was turned slightly to the contralateral side for the BPB.

**Figure 2:**
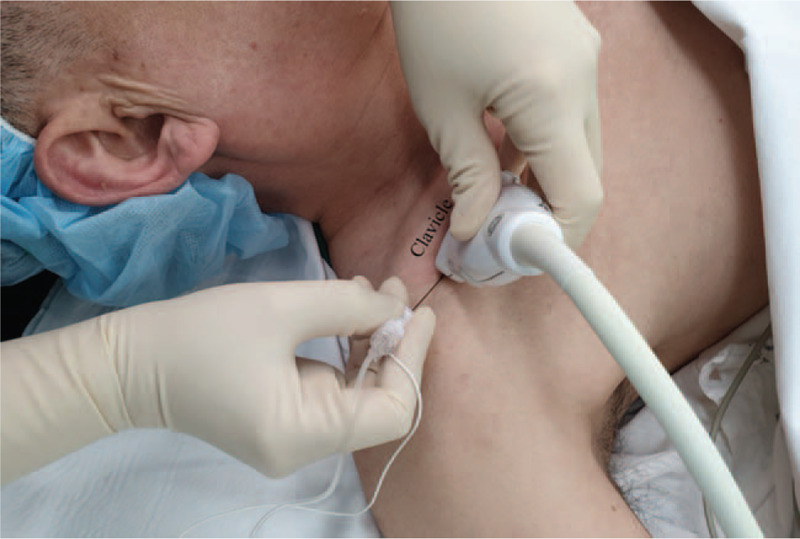
The position of the transducer in the CC approach. CC = costoclavicular.

The BPBs were performed under US guidance, and strict aseptic precautions were followed. A 22-gauge, 80-mm nerve stimulating needle (Uniplex, Pajunk GmbH Medizintechnologie, Geisingen, Germany), and a US system (GE LOGIQ P9, GE Healthcare, Little Chalfont, UK) with a high-frequency (L 4–12 MHz) linear array transducer was used for the BPB in both groups. A nerve stimulator was used to avoid nerve injury along the needling pathway, not for the sensory or motor evaluation. The transducer was positioned immediately below the midpoint of the clavicle and over the medial infraclavicular fossa (Fig. 2). The transducer was also tilted slightly cephalad to direct the US beam towards the CC space. In the CC space, the axillary artery was identified underneath the subclavius muscle. The US image was optimized until all 3 cords of the brachial plexus were visualized laterally to the axillary artery in one plane.

All blocks were performed under LA infiltration (2 mL of 1% lidocaine). The block needle was inserted in-plane and from a lateral-to-medial direction. The total volume of the LA mixture was 25 mL (10 mL of 2% lidocaine mixed with 10 mL of 0.75% ropivacaine and 5 mL of normal saline) in each group. The LA was injected in 2 to 3 mL increments after intermittent negative aspiration under direct US visualization of the LA spread. If paresthesia was induced during the procedure, the needle was withdrawn by 2 to 3 mm. The anesthesiologist then ensured that paresthesia was not induced before injecting the LA. The needle tip was always visualized before the LA injection. The US screen was positioned such that it was not visible to the patients in either group.^[8]^

**Figure 3:**
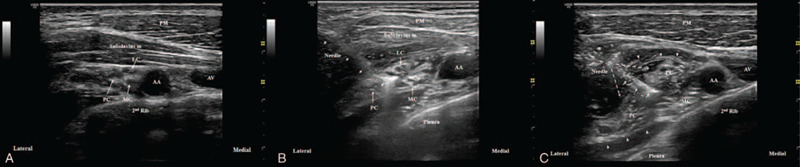
Single- (SI) versus triple-injection (TI) costoclavicular approach methods. (A) Pre-block, all 3 cords of the brachial plexus can be visualized laterally to the axillary artery. (B) In the SI group, the block needle is advanced to the center of the 3 cords. After the correct needle tip position is confirmed, 25 mL of the local anesthetic (LA) is slowly injected. The short white arrows indicate the block needle shaft. (C) In the TI group, the block needle is advanced to the medial cord, lateral cord, and posterior cord. One-third of the LA volume is injected in each of the cords in order. The short white arrows indicate the first block needle. The white dotted arrow indicates the direction of the second and third needle (needles not visible). The white arrowhead indicates the neural sheath, which is stretched outside due to the spread of the LA. AA = axillary artery, AV = axillary vein, LC = lateral cord, MC = medial cord, PC = posterior cord, PM = pectoralis major muscle, subclavius m = subclavius muscle.

In the SI group, after the skin puncture, the block needle was advanced to the brachial plexus sheath. After the sheath was penetrated, a small amount (0.5–1 mL) of 0.9% normal saline was then incrementally injected to “open” the perineural space until the needle tip was positioned at the center of the cord cluster.^[9]^ After the correct needle tip position was confirmed, 25 mL of the LA was slowly injected. The spread of the LA from the center of the 3 cords was observed. There was no noticeable swelling of the cords of the brachial plexus in the US image.^[6,7]^ In the TI group, after the skin puncture, the block needle was advanced to the medial cord similar to the description above (hydrodissection). One-third of the LA volume was then injected into the medial cord. The needle tip was then redirected to the lateral and posterior cords, with one-third of the LA volume being slowly injected in each cord. Subsequent advancement of the needle was preceded by withdrawal of the needle by approximately 10 to 15 mm; however, the needle was not withdrawn to the subcutaneous tissue. The spread of the LA around each of the 3 cords was observed (Fig. 3).

### 2.3 Evaluations

Imaging time (defined as the time interval between the contact of the US transducer with the patient and the acquisition of a satisfactory image) and the needling time (defined as the time interval between the advancement of the needle to the skin and the termination of the LA injection through the block needle; the needling time was applied after the temporal interval [1–2 minutes] from the skin weal) were recorded. Thus, performance time was defined as the sum of the imaging and needling times.

Subsequently, BPB was evaluated from immediately after the LA injection every 5 minutes for up to 30 minutes by a single blinded observer. The sensory block was evaluated using an alcohol swab on the dermatomes of the ulnar (fifth finger), median (palmar aspect of the second finger), radial (dorsum of the hand between the thumb and second finger), and musculocutaneous (lateral aspect of the forearm) nerves.^[6]^ The patients quantified the level of the sensory block using an 11-point scale (10 = normal sensation, 0 = no sensation to cold). A complete sensory block was defined by a score of 0 in each nerve dermatome. The motor block was evaluated using a 3-point scale where 2 signified no block; 1, paresis, that is, reduced force compared with the contralateral arm; and 0, paralysis, that is, incapacity to overcome gravity, which was applied to the whole arm.^[6]^ Accordingly a complete motor block was defined by a score of 0. Onset time was defined as the time required to obtaining full sensory and motor block of the median, ulnar, radial, and musculocutaneous nerves.^[10]^ The cases where even one nerve was missed were excluded from the calculation of the onset time. After completing this evaluation, the patient was moved to the operating room for the surgery.

When a patient requested sedation during the surgery, 2 to 5 mg midazolam was administered based on the decision of the anesthesiologist, who was blinded to the group allocations.

At the end of the surgery, anesthesia grade was assessed using a 4-point scale, as follows: excellent = when the surgery was finished with only a brachial plexus block; good = complete analgesia, but the patient complained about their position necessitating intravenous (IV) medication (<100 μg fentanyl and 5 mg midazolam); insufficient = when IV medication of ≥100 μg fentanyl and 5 mg midazolam or propofol infusion (25–80 μg/kg/min) or an additional local injection at the operative site was required, but the surgery was finished successfully; and failure = when general anesthesia was required to complete the surgery.^[10]^

Presence of hemidiaphragmatic paralysis (HDP), detected by comparison of the pre- and postoperative chest radiographs, and presence of other complications (e.g., hematoma formation, Horner syndrome, hoarseness, respiratory distress, neurological complications, nausea, and vomiting) were evaluated in the post-anesthetic care unit by an independent observer who was blinded to the group allocations. The HDP grade was assessed, as follows: normal = no diaphragmatic paralysis; partial = elevation of the diaphragm ≤4 cm above its preoperative position; and complete = elevation of the diaphragm >4 cm above its preoperative position.^[11]^ The primary outcome variable was the rate of blockage of all 4 nerves. The secondary outcome variables were the performance time, onset time, and anesthesia grade.

### 2.4 Statistical analysis

In a preliminary study, all 4 nerves were blocked in 7 out of 10 SI-treated patients, and 9 out of 10 TI-treated patients. Thirty-four patients were required per group for an α value of 0.05 and a power of 90%. Therefore, 68 patients were recruited. The results are presented as mean ± standard deviation, unless otherwise indicated. The statistical analysis was performed using SPSS (SPSS, version 19.0, Chicago, IL). The chi-square or Fisher exact test was used to analyze categorical data, and the Student unpaired *t* test was used to compare the continuous data. A *P-*value <.05 was considered statistically significant.

## 3 Results

Patient demographic data are shown in Table 1. No significant differences were observed between the 2 groups.

**Table 1:**
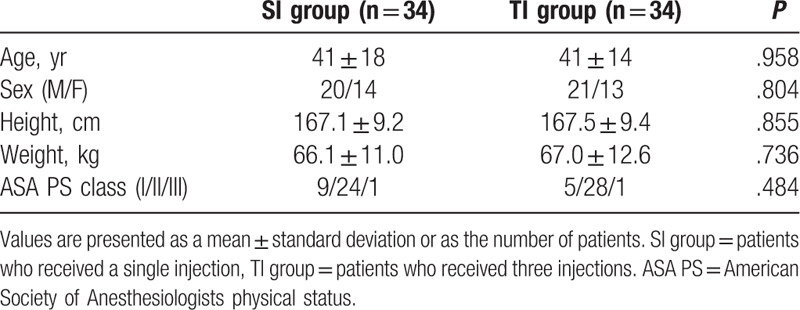
Patient characteristics of the 2 groups.

Data regarding US-guided infra-BPB are shown in Table 2. A skin puncture was performed once in both groups, except for 1 case in the TI group, where 2 skin punctures were performed due to an out-of-plane injection in 1 cord (this is further explained in the Discussion section below). The performance time of the TI group and SI group was similar. The block onset time of the TI group was not significantly different from that of the SI group. However, the rate of blockage of all 4 nerves was significantly higher in the TI group than in the SI group.

**Table 2:**
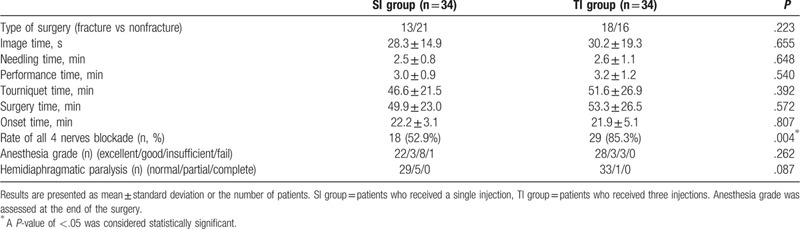
Ultrasound-guided infraclavicular brachial plexus block data.

**Figure 4:**
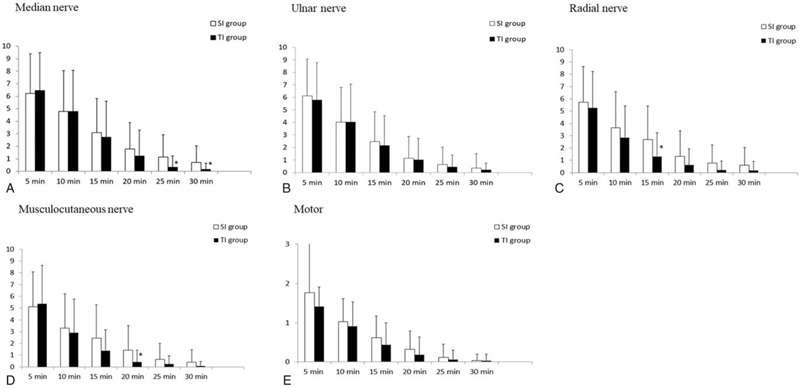
Time courses of the sensory and motor tests for the median, ulnar, radial, and musculocutaneous nerves. The vertical axis represents an 11-point scale (10 = normal sensation, 0 = no sensation to cold) (A–D), or a 3-point scale (2 = no block, 1 = paresis, 0 = paralysis) (E). Data are presented as the mean. The bar represents the standard deviation. ∗A *P*-value of <.05 was considered statistically significant.

The proportion of patients with complete sensory block and complete motor block at each evaluation time up to 30 minutes after the block was similar in both groups, except for the patients with the radial nerve block at 15 minutes, those with the musculocutaneous nerve block at 20 minutes, and those with the median nerve at 25 and 30 minutes (Fig. 4).

No vascular or pleural punctures occurred during the procedures. Other complications were ptosis (1 case), and paresthesia (2 cases) in the SI group and nausea (1 case), and hoarseness (2 cases) in the TI group. Complete recovery of sensory and motor function was confirmed in all patients. No neurologic complications were reported at the 1-week follow-up.

## 4 Discussion

The primary finding of this study was that the TI group increased the consistency of infraclavicular BPB in terms of the rate of blockage of all 4 nerves compared with the SI group, without an increase in the procedure time using the same volume (25 mL) of the LA for US-guided infraclavicular BPBs with a CC approach.

Karmakar et al^[6,7]^ recently introduced the CC approach with the aim of targeting the CC space where the 3 cords are tightly clustered together. While effective surgical anesthesia was provided, the rate of blockage of all 4 nerves was about 50% 30 minutes after the block, which was similar to the results of the SI group in this study (52.9%).

In our study, the rates of “excellent” anesthesia grade (when surgery was finished with only a BPB) were similar in the 2 groups (SI group 64.7% vs TI group 82.4%, *P* = .99). But we primarily focused our study on the successful rate of all 4 nerves blockage because failure in blocking 1 nerve completely can lower the anesthesia grade if surgery is performed in an area innervated by an incompletely blocked nerve.^[12]^ Furthermore, it was thought to be more meaningful than shortening the onset time.^[10]^

Layera et al^[6–8]^ recently compared a single injection technique with the double injections technique using the CC approach. In their study, the double injection technique displayed a shorter block onset time. However, this might be partially explained by a relatively larger LA volume than the amount used in the first CC approach (35 mL). An increase in the volume can enhance the block quality, but the probability of LA toxicity can also increase.^[12]^ In the current study, we used triple injections to target specific cords. However, the LA was divided so that only one-third of the total volume was injected in each of the cords.

Figure 4 shows that the median, radial, and musculocutaneous nerves were blocked faster at certain time intervals in the TI group. However, this did not lead to a decrease in the onset time. The median nerve emerges from the medial and the lateral cords, the radial nerve from the posterior cord, and the musculocutaneous nerve from the lateral cord, so triple injections seem to be effective in ensuring the even distribution of LA to each of the 3 cords.

In the conventional approach, all 3 cords are rarely visualized in a single sagittal US scan.^[7]^ In all cases in this study, we saw 3 cords in 1 US plane. Therefore, we believe that the CC approach is advantageous in the clinical setting. However, it can be challenging to advance the needle to the desired site. In 1 female patient (159 cm tall and weighing 39 kg [underweight]) in the TI group, the needle could not be advanced to the medial and lateral cords using the in-plane technique due to the angle. Therefore, we used the out-of-plane technique, and the needle could be inserted at the center of the medial cord and the lateral cord. The LA spread towards these 2 cords was confirmed by US. Subsequently, we could advance the needle to the posterior cord using the in-plane technique. The out-of-plane technique can be principally used in situations where the in-plane technique is challenging or the needle direction is not clear.^[13]^

When a patient is overweight or muscular, it can be challenging to view the whole shaft of the needle when in deep tissue. Therefore, the results could differ depending on the proficiency of the clinician. We attempted to view the needle tip and confirm the needle advancement with tissue movement using the US image.^[13,14]^ Needle advancement or LA injections without adequate needle tip visualization can cause unintentional vascular, neural, or visceral injuries.^[14]^

In the pre-block US view, 3 cords were observed as 1 compact neural tissue similar to divisions in the supraclavicular area (Fig. 3A). However, after the LA was injected, the 3 cords rose against the backdrop of anechoic LA spreading. Therefore, after the needle had penetrated the nerve sheath, we used hydrodissection for advancing the needle to the center of the 3 cords.

In the CC approach, the cephalic vein can be visualized during the needle pathway. Therefore, clinicians must be careful due to the possibility of a vascular puncture.^[3]^ If the transducer comes out of the CC space, into a slightly inferior direction, the clinician will come across a few small vessels on the needle pathway. Two cases in the SI group had blood flow where the first transducer was laid, so we tilted the US transducer slightly cephalad in order to provide an adequate US view of the CC space, and there were no blood aspirations in 2 cases.^[3]^

More needle passes were required in the TI group as expected. We did not evaluate the procedure-related pain score immediately after the block, but no one specifically complained of discomfort or pain in either group. In the TI group, the skin was only punctured once, and the needle was not taken to the subcutaneous tissue but withdrawn about 10 to 15 mm before the needle was re-advanced.

The anesthesiologist who performed all blocks in the present study was not blinded to the group allocations. However, the sensory-motor test evaluations were performed by an independent blinded observer. Therefore, we believe that unintentional bias had little impact on the overall results.^[12]^

We did not use a nerve stimulator as a tool to confirm each cord, and the block was only performed on the basis of the anatomical topography of the CC space.^[6,15]^ Using the study by Chang et al,^[12]^ we assumed that there is a consistent anatomical relationship between the 3 cords and the axillary artery (the 3 cords are in a fixed position in the CC space). We believe that a more accurate block at tolerable LA doses is possible if anatomical and technical factors are adequately considered.

We did not want to increase the volume of LA for improving the block quality in this study, so we chose the TI approach that required some technical proficiency. The single injection CC approach of infraclavicular BPB is a simple and easy technique for hand surgery. And we can expect the good results if we increase the volume of LA of SI. But we think further research is needed if we want to prove whether SI with ≥30 mL LA volume is comparative to the TI with 25 mL in increasing the block consistency.

In conclusion, the TI of CC approach increased the consistency of US-guided infraclavicular BPB in terms of the rate of blocking all 4 nerves without increasing the procedure time, despite administration of the same volume (25 mL) of LA.

## Author contributions

**Conceptualization:** Hyeon Ju Shin.

**Data curation:** Doo Yeon Go, Hye Won Shin.

**Formal analysis:** Wol Seon Jung.

**Investigation:** Mi Geum Lee, Yun Suk Choi, Hyeon Ju Shin.

**Methodology:** Sung Uk Choi.

**Supervision:** Hyeon Ju Shin.

**Writing – original draft:** Mi Geum Lee.

**Writing – review & editing:** Mi Geum Lee, Hyeon Ju Shin.
